# (*E*)-2-{Eth­yl[4-(4-nitro­phenyl­diazen­yl)phen­yl]amino}ethyl anthracene-9-carboxyl­ate

**DOI:** 10.1107/S1600536808004108

**Published:** 2008-02-15

**Authors:** Mark A. Rodriguez, Thomas Zifer, Andrew L. Vance, Bryan M. Wong, Francois Leonard

**Affiliations:** a PO Box 5800, MS 1411, Sandia National Laboratories, Albuquerque, NM 87185, USA; b PO Box 969, MS 9403, Sandia National Laboratories, Livermore, CA 94551, USA; c PO Box 969, MS 9161, Sandia National Laboratories, Livermore, CA 94551, USA

## Abstract

The crystal structure of the title compound, C_31_H_26_N_4_O_4_, displays a *trans* conformation for the nitro­phenyl­diazenyl portion of the mol­ecule. Packing diagrams indicate that weak C—H⋯O hydrogen bonds, likely associated with a strong dipole moment present in the mol­ecule, dictate the arrangement of mol­ecules in the crystal structure.

## Related literature

Simmons *et al.* (2007 ▶) describe the use of the title compound in the fabrication of carbon nanotubes with optically modulated electronic conduction. Sekkat *et al.* (1992 ▶) document the use of Disperse Red 1 for reversible photoisomerization in thin films.

For related literature, see: Atassi *et al.* (1998 ▶); Becke (1993 ▶).
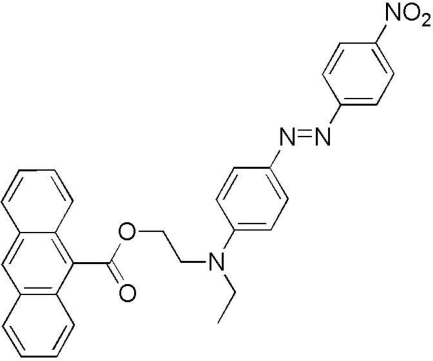


## Experimental

### 

#### Crystal data

C_31_H_26_N_4_O_4_
*M* *_r_* = 518.56
Triclinic, 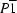

*a* = 9.3161 (9) Å
*b* = 10.6586 (10) Å
*c* = 13.5328 (13) Å
α = 101.134 (3)°
β = 104.667 (4)°
γ = 99.779 (3)°
*V* = 1241.2 (2) Å^3^
*Z* = 2
Mo *K*α radiation
μ = 0.09 mm^−1^
*T* = 173 (2) K
0.20 × 0.08 × 0.06 mm

#### Data collection

Bruker SMART CCD area-detector diffractometer
Absorption correction: multi-scan (*SADABS*; Sheldrick, 1999 ▶) *T*_min_ = 0.982, *T*_max_ = 0.994
9650 measured reflections
4824 independent reflections
2786 reflections with *I* > 2σ(*I*)
*R*_int_ = 0.050

#### Refinement

*R*[*F* ^2^ > 2σ(*F* ^2^)] = 0.066
*wR*(*F* ^2^) = 0.123
*S* = 1.02
4824 reflections
353 parameters
H-atom parameters constrained
Δρ_max_ = 0.21 e Å^−3^
Δρ_min_ = −0.21 e Å^−3^

### 

Data collection: *SMART* (Bruker, 1998 ▶); cell refinement: *SMART*; data reduction: *SAINT-Plus* (Bruker, 2001 ▶); program(s) used to solve structure: *SHELXTL* (Sheldrick, 2008 ▶); program(s) used to refine structure: *XSHELL* (Bruker, 2000 ▶); molecular graphics: *XSHELL* and *Mercury* (Macrae *et al.*, 2006 ▶); software used to prepare material for publication: *SHELXTL*.

## Supplementary Material

Crystal structure: contains datablocks I, global. DOI: 10.1107/S1600536808004108/fl2188sup1.cif

Structure factors: contains datablocks I. DOI: 10.1107/S1600536808004108/fl2188Isup2.hkl

Additional supplementary materials:  crystallographic information; 3D view; checkCIF report

## Figures and Tables

**Table 1:** Hydrogen-bond geometry (Å, °)

*D*—H⋯*A*	*D*—H	H⋯*A*	*D*⋯*A*	*D*—H⋯*A*
C1—H1⋯O3^i^	0.95	2.56	3.230 (4)	128
C3—H3⋯O4^i^	0.95	2.65	3.570 (4)	163
C16—H16*B*⋯O4^i^	0.99	2.61	3.462 (4)	144
C21—H21⋯O2^ii^	0.95	2.31	3.176 (4)	152

Symmetry codes: (i) 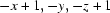; (ii) 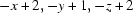.

**Fig. 1.**
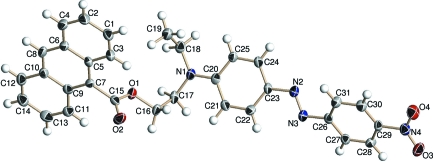
The molecular structure of (I), with atom labels and 50% probability displacement ellipsoids for non-H atoms.

**Fig. 2.**
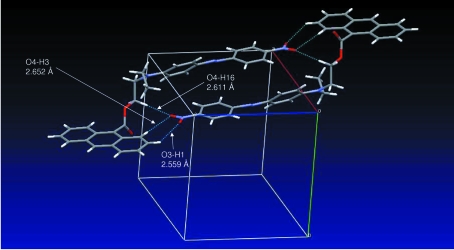
Packing of (I) as viewed down the a axis illustrating weak C—H···O hydrogen bonding associated with terminal O atoms O3 and O4. The nitroasobenzene portion of (I) resides near the a-c plane of the unit cell.

**Fig. 3.**
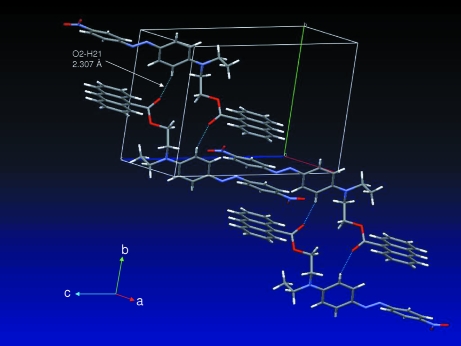
Packing diagram of (I) viewed down the a axis illustrating weak hydrogen bonding between the carbonyl oxygen (O2) and the neighboring molecule of (I). This interaction dictates the packing behavior of (I) along the b axis.

## supplementary crystallographic information

### Comment

Figure 1 shows an atomic displacement ellipsoid plot of the title compound (I). (I) is a merger of 9-anthracenecarboxylic acid and 4-[*N*-(2-hydroxyethyl)-*N*-ethyl]-amino-4'-nitroazobenzene which is better known as Disperse Red 1 or (DR1). In (I) the azobenzene-based DR1 takes on the *trans* conformational state. Atassi *et al.* (1998) has documented photoisomerization to a *cis* conformation under UV light with decay back to the equilibrium *trans* species upon removal of the UV stimulus. (I) has three terminal oxygen atoms: O2, O3 and O4. All three of these atoms display double bonds, O2 being a remnant of anthracene and O3 and O4 at the termination of the nitroazobenzene. The C15=O2 bond length is 1.207 (3)Å while O3=N4 and O4=N4 are slightly longer at 1.230 (3) and 1.229 (3) Å, respectively. All other bond lengths (C—C, C—N, N=N, and C—O) in (I) were consistent with expected values.

Figure 2 shows a packing arrangement of two molecules of (I). The two molecules are related by inversion, consistent with the P-1 space group and the nitroazobenzene portion of (I) is positioned close to the a-c plane of the unit cell. Weak C—H···O hydrogen bonds are observed from O3 to H1—C1 of a neighboring molecule, with an intermolecular O3···H1 distance of 2.559 Å. Likewise, O4 shows similar weak hydrogen bonding to H3—C3 and H16—C16 of a neighboring molecule with intermolecular distances of 2.652 and 2.611 Å, respectively. These weak C—H···O hydrogen bonds generate a supramolecular head-to-tail dimer *via* the nitro groups that terminate each molecule. Atassi, *et al.* (1998) has calculated the dipole moment for the *trans* form of DR1 to be about 9D. Since compound (I) contains the DR1 molecule, it is reasonable to presume a comparable dipole presence for (I). Our calculations of the geometry and dipole moment for compound (I) using a three-parameter hybrid functional (B3LYP) with the 6–311 G(d,p) basis set (Becke, 1993) yielded a value of 11.8 D. This relatively strong dipole likely plays a role in the head-to-tail alignment of the molecules as viewed in Figure 2.

Figure 3 shows a packing diagram for (I) which illustrates the supramolecular interactions along the *b* axis of the unit cell. Again we can see the inversion symmetry for the two molecules of (I) and see that the carbonyl O2 atom is coordinated to H21—C21 of a neighboring molecule with an intermolecular distance of 2.307 Å. This is shorter than for interactions observed in figure 2 and likely indicates a more rigid C—H···O interaction along the *b* axis.

### Experimental

The title compound was obtained using the published synthetic procedure of Simmons, *et al.* (2007). The product was synthesized from 9-anthracenecarboxylic acid and Disperse red 1 *via* a dicylcohexylcarbodiimide esterification in anhydrous dichloromethane. Following purification by silica gel chromatography with chloroform eluent, the dark red powder was characterized by 1*H*-NMR, UV/Vis and FTIR. Crystals were obtained by re-crystallization from acetonitrile/ethanol.

### Crystal data

**Table:**

C_31_H_26_N_4_O_4_	*Z* = 2
*M**_r_* = 518.56	*F*_000_ = 544
Triclinic, *P*1	*D*_x_ = 1.388 Mg m^−^^3^
Hall symbol: -P 1	Mo *K*α radiation λ = 0.71073 Å
*a* = 9.3161 (9) Å	Cell parameters from 100 reflections
*b* = 10.6586 (10) Å	θ = 1.6–26.0º
*c* = 13.5328 (13) Å	µ = 0.09 mm^−^^1^
α = 101.134 (3)º	*T* = 173 (2) K
β = 104.667 (4)º	Irregular, orange
γ = 99.779 (3)º	0.20 × 0.08 × 0.06 mm
*V* = 1241.2 (2) Å^3^	

### Data collection

**Table:**

Bruker SMART CCD area-detector diffractometer	4824 independent reflections
Radiation source: fine-focus sealed tube	2786 reflections with *I* > 2σ(*I*)
Monochromator: graphite	*R*_int_ = 0.050
Detector resolution: 0 pixels mm^-1^	θ_max_ = 26.0º
*T* = 173(2) K	θ_min_ = 1.6º
phi and ω scans	*h* = −11→11
Absorption correction: multi-scan(SADABS; Sheldrick, 1999)	*k* = −13→13
*T*_min_ = 0.982, *T*_max_ = 0.994	*l* = −16→16
9650 measured reflections	

### Refinement

**Table:**

Refinement on *F*^2^	Secondary atom site location: difference Fourier map
Least-squares matrix: full	Hydrogen site location: inferred from neighbouring sites
*R*[*F*^2^ > 2σ(*F*^2^)] = 0.066	H-atom parameters constrained
*wR*(*F*^2^) = 0.123	*w* = 1/[σ^2^(*F*_o_^2^) + (0.0333*P*)^2^] where *P* = (*F*_o_^2^ + 2*F*_c_^2^)/3
*S* = 1.02	(Δ/σ)_max_ = 0.001
4824 reflections	Δρ_max_ = 0.21 e Å^−^^3^
353 parameters	Δρ_min_ = −0.21 e Å^−^^3^
Primary atom site location: structure-invariant direct methods	Extinction correction: none

### Special details

**Table:**

Geometry. All e.s.d.'s (except the e.s.d. in the dihedral angle between two l.s. planes) are estimated using the full covariance matrix. The cell e.s.d.'s are taken into account individually in the estimation of e.s.d.'s in distances, angles and torsion angles; correlations between e.s.d.'s in cell parameters are only used when they are defined by crystal symmetry. An approximate (isotropic) treatment of cell e.s.d.'s is used for estimating e.s.d.'s involving l.s. planes.
Refinement. Refinement of *F*^2^ against ALL reflections. The weighted *R*-factor *wR* and goodness of fit *S* are based on *F*^2^, conventional *R*-factors *R* are based on *F*, with *F* set to zero for negative *F*^2^. The threshold expression of *F*^2^ > σ(*F*^2^) is used only for calculating *R*-factors(gt) *etc*. and is not relevant to the choice of reflections for refinement. *R*-factors based on *F*^2^ are statistically about twice as large as those based on *F*, and *R*- factors based on ALL data will be even larger.

### Fractional atomic coordinates and isotropic or equivalent isotropic displacement parameters (Å^2^)

**Table:**

	*x*	*y*	*z*	*U*_iso_*/*U*_eq_	
C1	0.4311 (4)	0.4301 (3)	1.1818 (2)	0.0357 (8)	
H1	0.3552	0.3878	1.1167	0.043*	
C2	0.3871 (4)	0.4647 (3)	1.2740 (2)	0.0380 (8)	
H2	0.2823	0.4455	1.2705	0.046*	
C3	0.5796 (3)	0.4565 (3)	1.1849 (2)	0.0314 (8)	
H3	0.6066	0.4332	1.1218	0.038*	
C4	0.4936 (3)	0.5249 (3)	1.3672 (2)	0.0330 (8)	
H4	0.4624	0.5486	1.4285	0.040*	
C5	0.6965 (3)	0.5190 (3)	1.2820 (2)	0.0256 (7)	
C6	0.6513 (3)	0.5536 (3)	1.3752 (2)	0.0267 (7)	
C7	0.8515 (3)	0.5491 (3)	1.2887 (2)	0.0233 (7)	
C8	0.7638 (3)	0.6146 (3)	1.4708 (2)	0.0295 (8)	
H8	0.7339	0.6371	1.5328	0.035*	
C9	0.9635 (3)	0.6111 (3)	1.3845 (2)	0.0262 (7)	
C10	0.9183 (3)	0.6438 (3)	1.4782 (2)	0.0271 (7)	
C11	1.1225 (3)	0.6452 (3)	1.3946 (2)	0.0308 (8)	
H11	1.1560	0.6250	1.3339	0.037*	
C12	1.0315 (4)	0.7073 (3)	1.5759 (2)	0.0363 (8)	
H12	1.0017	0.7292	1.6380	0.044*	
C13	1.2262 (4)	0.7058 (3)	1.4892 (3)	0.0385 (9)	
H13	1.3313	0.7279	1.4938	0.046*	
C14	1.1804 (4)	0.7369 (3)	1.5816 (3)	0.0404 (9)	
H14	1.2548	0.7785	1.6476	0.049*	
C15	0.8955 (3)	0.5179 (3)	1.1890 (2)	0.0262 (7)	
C16	0.9915 (3)	0.3710 (3)	1.0822 (2)	0.0283 (7)	
H16A	1.0823	0.4372	1.0856	0.034*	
H16B	0.9086	0.3672	1.0185	0.034*	
C17	1.0292 (3)	0.2372 (3)	1.0778 (2)	0.0306 (8)	
H17A	1.1023	0.2286	1.0364	0.037*	
H17B	1.0808	0.2334	1.1503	0.037*	
C18	0.8293 (4)	0.0686 (3)	1.1033 (2)	0.0326 (8)	
H18A	0.9052	0.0919	1.1739	0.039*	
H18B	0.8035	−0.0283	1.0777	0.039*	
C19	0.6864 (4)	0.1132 (3)	1.1141 (2)	0.0384 (9)	
H19A	0.7124	0.2081	1.1454	0.058*	
H19B	0.6428	0.0670	1.1595	0.058*	
H19C	0.6117	0.0934	1.0443	0.058*	
C20	0.8434 (3)	0.0764 (3)	0.9246 (2)	0.0252 (7)	
C21	0.9076 (3)	0.1361 (3)	0.8559 (2)	0.0271 (7)	
H21	0.9850	0.2148	0.8846	0.032*	
C22	0.8607 (3)	0.0830 (3)	0.7490 (2)	0.0274 (7)	
H22	0.9083	0.1238	0.7053	0.033*	
C23	0.7442 (3)	−0.0300 (3)	0.7038 (2)	0.0248 (7)	
C24	0.6742 (3)	−0.0860 (3)	0.7695 (2)	0.0275 (7)	
H24	0.5922	−0.1614	0.7394	0.033*	
C25	0.7210 (3)	−0.0349 (3)	0.8769 (2)	0.0259 (7)	
H25	0.6704	−0.0750	0.9195	0.031*	
C26	0.6993 (3)	−0.0968 (3)	0.4302 (2)	0.0248 (7)	
C27	0.7983 (3)	−0.1047 (3)	0.3689 (2)	0.0271 (7)	
H27	0.9051	−0.0733	0.4009	0.033*	
C28	0.7414 (3)	−0.1583 (3)	0.2614 (2)	0.0270 (7)	
H28	0.8081	−0.1663	0.2190	0.032*	
C29	0.5850 (3)	−0.1999 (3)	0.2170 (2)	0.0255 (7)	
C30	0.4844 (3)	−0.1891 (3)	0.2755 (2)	0.0303 (8)	
H30	0.3775	−0.2172	0.2427	0.036*	
C31	0.5424 (3)	−0.1365 (3)	0.3829 (2)	0.0298 (7)	
H31	0.4751	−0.1274	0.4246	0.036*	
N1	0.8986 (3)	0.1256 (2)	1.03191 (18)	0.0281 (6)	
N2	0.6851 (3)	−0.0883 (2)	0.59401 (18)	0.0278 (6)	
N3	0.7671 (3)	−0.0435 (2)	0.54054 (18)	0.0281 (6)	
N4	0.5237 (3)	−0.2560 (2)	0.10309 (19)	0.0333 (7)	
O1	0.9436 (2)	0.40590 (19)	1.17640 (14)	0.0297 (5)	
O2	0.8885 (3)	0.5849 (2)	1.12621 (17)	0.0432 (6)	
O3	0.6111 (3)	−0.2485 (2)	0.04881 (16)	0.0490 (7)	
O4	0.3869 (3)	−0.3075 (2)	0.06570 (16)	0.0461 (6)	

### Atomic displacement parameters (Å^2^)

**Table:**

	*U*^11^	*U*^22^	*U*^33^	*U*^12^	*U*^13^	*U*^23^
C1	0.030 (2)	0.0329 (19)	0.036 (2)	0.0005 (16)	0.0081 (16)	−0.0016 (16)
C2	0.030 (2)	0.035 (2)	0.046 (2)	0.0021 (16)	0.0162 (17)	0.0015 (17)
C3	0.036 (2)	0.0261 (18)	0.0289 (18)	0.0055 (15)	0.0106 (16)	−0.0005 (14)
C4	0.036 (2)	0.0326 (19)	0.035 (2)	0.0098 (16)	0.0191 (17)	0.0063 (16)
C5	0.0308 (19)	0.0178 (16)	0.0267 (18)	0.0049 (14)	0.0099 (15)	0.0010 (13)
C6	0.0305 (19)	0.0233 (17)	0.0282 (18)	0.0062 (14)	0.0133 (15)	0.0046 (14)
C7	0.0281 (18)	0.0186 (16)	0.0264 (17)	0.0059 (13)	0.0135 (14)	0.0054 (13)
C8	0.040 (2)	0.0282 (18)	0.0238 (17)	0.0098 (15)	0.0150 (16)	0.0049 (14)
C9	0.0315 (19)	0.0203 (16)	0.0285 (18)	0.0071 (14)	0.0084 (15)	0.0096 (14)
C10	0.0320 (19)	0.0227 (17)	0.0256 (18)	0.0058 (14)	0.0084 (15)	0.0045 (14)
C11	0.0299 (19)	0.0324 (19)	0.0326 (19)	0.0089 (15)	0.0110 (16)	0.0100 (15)
C12	0.043 (2)	0.036 (2)	0.0251 (18)	0.0072 (17)	0.0068 (16)	0.0028 (15)
C13	0.029 (2)	0.036 (2)	0.047 (2)	0.0041 (16)	0.0076 (17)	0.0102 (17)
C14	0.044 (2)	0.035 (2)	0.032 (2)	0.0020 (17)	0.0012 (17)	0.0036 (16)
C15	0.0232 (18)	0.0212 (17)	0.0312 (19)	0.0026 (14)	0.0074 (15)	0.0027 (14)
C16	0.0292 (18)	0.0356 (19)	0.0184 (16)	0.0067 (15)	0.0081 (14)	0.0021 (14)
C17	0.0261 (18)	0.0376 (19)	0.0230 (17)	0.0103 (15)	0.0047 (14)	−0.0035 (15)
C18	0.043 (2)	0.0314 (19)	0.0253 (18)	0.0130 (16)	0.0100 (16)	0.0077 (15)
C19	0.046 (2)	0.035 (2)	0.036 (2)	0.0086 (17)	0.0176 (17)	0.0064 (16)
C20	0.0260 (18)	0.0265 (18)	0.0263 (18)	0.0137 (14)	0.0094 (15)	0.0052 (14)
C21	0.0234 (17)	0.0249 (17)	0.0292 (18)	0.0042 (14)	0.0072 (14)	0.0000 (14)
C22	0.0256 (18)	0.0321 (18)	0.0276 (18)	0.0088 (15)	0.0114 (14)	0.0076 (15)
C23	0.0248 (17)	0.0263 (17)	0.0204 (17)	0.0071 (14)	0.0052 (14)	0.0003 (13)
C24	0.0282 (18)	0.0263 (17)	0.0242 (17)	0.0053 (14)	0.0061 (14)	0.0007 (14)
C25	0.0292 (18)	0.0249 (17)	0.0242 (17)	0.0075 (14)	0.0095 (14)	0.0046 (14)
C26	0.0329 (19)	0.0179 (16)	0.0247 (17)	0.0092 (14)	0.0085 (15)	0.0048 (13)
C27	0.0248 (17)	0.0273 (17)	0.0256 (18)	0.0035 (14)	0.0053 (14)	0.0033 (14)
C28	0.0268 (18)	0.0322 (18)	0.0250 (18)	0.0079 (15)	0.0095 (14)	0.0104 (14)
C29	0.0332 (19)	0.0233 (17)	0.0178 (16)	0.0077 (14)	0.0045 (14)	0.0032 (13)
C30	0.0271 (18)	0.0328 (19)	0.0285 (18)	0.0077 (15)	0.0050 (15)	0.0056 (15)
C31	0.0293 (19)	0.0346 (19)	0.0268 (18)	0.0101 (15)	0.0096 (15)	0.0070 (15)
N1	0.0309 (16)	0.0331 (15)	0.0189 (14)	0.0075 (12)	0.0072 (12)	0.0028 (12)
N2	0.0308 (15)	0.0296 (15)	0.0245 (15)	0.0111 (12)	0.0093 (12)	0.0049 (12)
N3	0.0306 (16)	0.0308 (15)	0.0218 (14)	0.0083 (12)	0.0070 (12)	0.0037 (12)
N4	0.0376 (18)	0.0333 (16)	0.0253 (16)	0.0071 (14)	0.0045 (14)	0.0064 (13)
O1	0.0383 (13)	0.0302 (12)	0.0231 (12)	0.0128 (10)	0.0121 (10)	0.0043 (10)
O2	0.0657 (17)	0.0346 (14)	0.0456 (15)	0.0187 (12)	0.0329 (13)	0.0194 (12)
O3	0.0528 (16)	0.0625 (17)	0.0281 (13)	0.0052 (13)	0.0163 (12)	0.0045 (12)
O4	0.0316 (14)	0.0597 (16)	0.0327 (14)	0.0039 (12)	−0.0035 (11)	0.0011 (12)

### Geometric parameters (Å, °)

**Table:**

C1—C3	1.352 (4)	C18—N1	1.460 (3)
C1—C2	1.411 (4)	C18—C19	1.519 (4)
C1—H1	0.9500	C18—H18A	0.9900
C2—C4	1.350 (4)	C18—H18B	0.9900
C2—H2	0.9500	C19—H19A	0.9800
C3—C5	1.431 (4)	C19—H19B	0.9800
C3—H3	0.9500	C19—H19C	0.9800
C4—C6	1.421 (4)	C20—N1	1.373 (3)
C4—H4	0.9500	C20—C25	1.413 (4)
C5—C7	1.400 (4)	C20—C21	1.416 (4)
C5—C6	1.431 (4)	C21—C22	1.373 (4)
C6—C8	1.397 (4)	C21—H21	0.9500
C7—C9	1.399 (4)	C22—C23	1.392 (4)
C7—C15	1.500 (4)	C22—H22	0.9500
C8—C10	1.394 (4)	C23—C24	1.392 (4)
C8—H8	0.9500	C23—N2	1.418 (3)
C9—C11	1.429 (4)	C24—C25	1.376 (4)
C9—C10	1.433 (4)	C24—H24	0.9500
C10—C12	1.422 (4)	C25—H25	0.9500
C11—C13	1.354 (4)	C26—C27	1.391 (4)
C11—H11	0.9500	C26—C31	1.391 (4)
C12—C14	1.349 (4)	C26—N3	1.423 (3)
C12—H12	0.9500	C27—C28	1.381 (4)
C13—C14	1.418 (4)	C27—H27	0.9500
C13—H13	0.9500	C28—C29	1.384 (4)
C14—H14	0.9500	C28—H28	0.9500
C15—O2	1.207 (3)	C29—C30	1.378 (4)
C15—O1	1.341 (3)	C29—N4	1.463 (3)
C16—O1	1.457 (3)	C30—C31	1.379 (4)
C16—C17	1.519 (4)	C30—H30	0.9500
C16—H16A	0.9900	C31—H31	0.9500
C16—H16B	0.9900	N2—N3	1.275 (3)
C17—N1	1.457 (3)	N4—O4	1.229 (3)
C17—H17A	0.9900	N4—O3	1.230 (3)
C17—H17B	0.9900		
			
C3—C1—C2	120.9 (3)	N1—C18—C19	114.1 (3)
C3—C1—H1	119.5	N1—C18—H18A	108.7
C2—C1—H1	119.5	C19—C18—H18A	108.7
C4—C2—C1	120.2 (3)	N1—C18—H18B	108.7
C4—C2—H2	119.9	C19—C18—H18B	108.7
C1—C2—H2	119.9	H18A—C18—H18B	107.6
C1—C3—C5	120.9 (3)	C18—C19—H19A	109.5
C1—C3—H3	119.6	C18—C19—H19B	109.5
C5—C3—H3	119.6	H19A—C19—H19B	109.5
C2—C4—C6	121.2 (3)	C18—C19—H19C	109.5
C2—C4—H4	119.4	H19A—C19—H19C	109.5
C6—C4—H4	119.4	H19B—C19—H19C	109.5
C7—C5—C6	119.2 (3)	N1—C20—C25	122.4 (3)
C7—C5—C3	122.8 (3)	N1—C20—C21	121.0 (3)
C6—C5—C3	118.0 (3)	C25—C20—C21	116.6 (3)
C8—C6—C4	122.4 (3)	C22—C21—C20	121.7 (3)
C8—C6—C5	118.9 (3)	C22—C21—H21	119.2
C4—C6—C5	118.8 (3)	C20—C21—H21	119.2
C9—C7—C5	121.6 (3)	C21—C22—C23	120.8 (3)
C9—C7—C15	120.1 (3)	C21—C22—H22	119.6
C5—C7—C15	118.2 (3)	C23—C22—H22	119.6
C10—C8—C6	122.2 (3)	C22—C23—C24	118.4 (3)
C10—C8—H8	118.9	C22—C23—N2	124.4 (3)
C6—C8—H8	118.9	C24—C23—N2	117.1 (3)
C7—C9—C11	123.4 (3)	C25—C24—C23	121.6 (3)
C7—C9—C10	119.1 (3)	C25—C24—H24	119.2
C11—C9—C10	117.5 (3)	C23—C24—H24	119.2
C8—C10—C12	121.6 (3)	C24—C25—C20	120.8 (3)
C8—C10—C9	118.9 (3)	C24—C25—H25	119.6
C12—C10—C9	119.5 (3)	C20—C25—H25	119.6
C13—C11—C9	121.1 (3)	C27—C26—C31	120.1 (3)
C13—C11—H11	119.5	C27—C26—N3	116.6 (3)
C9—C11—H11	119.5	C31—C26—N3	123.3 (3)
C14—C12—C10	121.0 (3)	C28—C27—C26	120.0 (3)
C14—C12—H12	119.5	C28—C27—H27	120.0
C10—C12—H12	119.5	C26—C27—H27	120.0
C11—C13—C14	121.0 (3)	C27—C28—C29	118.5 (3)
C11—C13—H13	119.5	C27—C28—H28	120.8
C14—C13—H13	119.5	C29—C28—H28	120.8
C12—C14—C13	120.0 (3)	C30—C29—C28	122.6 (3)
C12—C14—H14	120.0	C30—C29—N4	118.6 (3)
C13—C14—H14	120.0	C28—C29—N4	118.8 (3)
O2—C15—O1	123.0 (3)	C29—C30—C31	118.5 (3)
O2—C15—C7	124.5 (3)	C29—C30—H30	120.8
O1—C15—C7	112.4 (3)	C31—C30—H30	120.8
O1—C16—C17	107.3 (2)	C30—C31—C26	120.3 (3)
O1—C16—H16A	110.3	C30—C31—H31	119.9
C17—C16—H16A	110.3	C26—C31—H31	119.9
O1—C16—H16B	110.3	C20—N1—C17	120.5 (2)
C17—C16—H16B	110.3	C20—N1—C18	121.5 (2)
H16A—C16—H16B	108.5	C17—N1—C18	118.0 (2)
N1—C17—C16	115.0 (2)	N3—N2—C23	113.5 (2)
N1—C17—H17A	108.5	N2—N3—C26	112.1 (2)
C16—C17—H17A	108.5	O4—N4—O3	122.8 (3)
N1—C17—H17B	108.5	O4—N4—C29	118.6 (3)
C16—C17—H17B	108.5	O3—N4—C29	118.6 (3)
H17A—C17—H17B	107.5	C15—O1—C16	115.4 (2)

### Hydrogen-bond geometry (Å, °)

**Table:**

*D*—H···*A*	*D*—H	H···*A*	*D*···*A*	*D*—H···*A*
C1—H1···O3^i^	0.95	2.56	3.230 (4)	128
C3—H3···O4^i^	0.95	2.65	3.570 (4)	163
C16—H16B···O4^i^	0.99	2.61	3.462 (4)	144
C21—H21···O2^ii^	0.95	2.31	3.176 (4)	152

Symmetry codes: (i) −*x*+1, −*y*, −*z*+1; (ii) −*x*+2, −*y*+1, −*z*+2.

## References

[bb1] Atassi, Y., Chauvin, J., Delaire, J. A., Delouis, J. F., Fanton-Maltey, I. & Nakatani, K. (1998). *Pure Appl. Chem.* **70**, 2157–2166.

[bb2] Becke, A. D. (1993). *J. Chem. Phys.* **98**, 5648–5652.

[bb3] Bruker (1998). *SMART.* Version 5.054. Bruker AXS Inc., Madison, Wisconsin, USA.

[bb4] Bruker (2000). *XSHELL.* Version 4.01. Bruker AXS Inc., Madison, Wisconsin, USA.

[bb5] Bruker (2001). *SAINT-Plus.* Version 6.02. Bruker AXS Inc., Madison, Wisconsin, USA.

[bb6] Macrae, C. F., Edgington, P. R., McCabe, P., Pidcock, E., Shields, G. P., Taylor, R., Towler, M. & van de Streek, J. (2006). *J. Appl. Cryst.* **39**, 453–457.

[bb7] Sekkat, Z., Morichere, D., Dumont, M., Loucif-Saibi, R. & Delaire, J. A. (1992). *J. Appl. Phys.* **71**, 1543–1545.

[bb8] Sheldrick, G. M. (1999). *SADABS.* Version 2.03. University of Göttingen, Germany.

[bb9] Sheldrick, G. M. (2008). *Acta Cryst.* A**64**, 112–122. 10.1107/S0108767307043930 18156677

[bb10] Simmons, J. M., In, I., Campbell, V. E., Mark, T. J., Leonard, F., Gopalan, P. & Eriksson, M. A. (2007). *Phys. Rev. Lett.* **98**, 086802. 10.1103/PhysRevLett.98.086802 17359117

